# CRISPR-CasRx Targeting LncRNA LINC00341 Inhibits Tumor Cell Growth *in vitro* and *in vivo*

**DOI:** 10.3389/fmolb.2021.638995

**Published:** 2021-03-09

**Authors:** Chunjing Li, Yu Cao, Li Zhang, Jierong Li, Jianfeng Wang, Yanfen Zhou, Huiling Wei, Mingjuan Guo, Liang Liu, Chunxiao Liu, Shilin Zhang, Guoqing Liu

**Affiliations:** ^1^ Affiliated Foshan Maternal and Child Healthcare Hospital, Southern Medical University, Foshan, China; ^2^ Department of Urology, Zhujiang Hospital of Southern Medical University, Guangzhou, China; ^3^ Ningxiang Hospital, Hunan University of Traditional Chinese Medicine, NingXiang, China; ^4^ The Second School of Clinical Medicine, Southern Medical University, Guangzhou, China

**Keywords:** long noncoding RNA, LINC00341, cancer, CRISPR-CasRx 3, CRISPR

## Abstract

CRISPR-CasRx technology provides a new and powerful method for studying cellular RNA in human cancer. Herein, the pattern of expression of long noncoding RNA 00341 (LINC00341) as well as its biological function in bladder cancer were studied using CRISPR-CasRx. qRT-PCR was employed to quantify the levels of expression of LINC00341 in tumor tissues along with the matched non-tumor tissues. sgRNA targeting LINC00341 or the sgRNA negative control were transiently transfected into the T24 as well as 5,637 human bladder cancer cell lines. CCK-8, ELISA as well as wound healing methods were employed to explore cell proliferation, apoptosis and migration, respectively. The tumorigenicity experiment in nude mice also performed to detect cell proliferation. The expression of p21, Bax as well as E-cadherin were assayed using western blot. The results demonstrated that LINC00341 was overexpressed in bladder cancer in contrast with the healthy tissues. The LINC00341 expression level in high-grade tumors was higher in contrast with that in low-grade tumors. The expression of linc00341 was higher relative to that of non-invasive tumors. In T24 as well as 5637-cell lines harboring LINC00341-sgRNA, inhibition of cell proliferation (*in vitro* and *in vivo*), elevated apoptosis rate and diminished migration ability. Moreover, silencing LINC00341 upregulated the expressions of p21, Bax as well as E-cadherin. Knockout of these genes could eliminate the phenotypic changes caused by sgRNA targeting LINC00341. Our data demonstrate that LINC00341 has a carcinogenic role in human bladder cancer.

## Introduction

Bladder cancer constitutes one of the most frequent malignancies in male urinary system worldwide and the ninth most frequent malignancy worldwide. Its most common histopathologic type is urothelial carcinoma. The genetic modulation is found to be responsible for the pathogenesis of bladder cancer, and the genetic mutations and epigenetic modifications that account to the development as well as progression of bladder cancer are to be identified (Chen et al., 2014; Wang et al., 2015).

Long non-coding RNAs (lncRNAs) comprise non-protein coding RNA transcripts that are larger than 200 nt in length (Gloss and Dinger, 2016). The investigations on lncRNAs have revealed that they can function as scaffolds or intermediators to form gene-gene networks and thus serve pivotal roles in the mediation of gene expression as well as disease progressions (Zhou et al., 2015). The dysregulation of lncRNAs is associated with the process of tumorigenesis, invasion, and metastasis, which makes them potential diagnostic and therapeutic biomarkers of human cancers (Hu et al., 2014; Jeong et al., 2016). LINC00341, a new long intergenic non-protein-coding RNA , whose role is unclear and no literatures have studied the biological role of this lncRNA in any diseases including cancers.

Herein, we established that LINC00341 is overexpressed in bladder cancer in contrast with the corresponding non-tumor bladder tissue. Moreover, the LINC00341 expression in high-grade tumors is elevated in contrast with low-grade tumors. LINC00341 RNA is more enriched in invasive tumors in contrast with non-invasive tumors. The knockdown of LINC00341 with CRISPR--CasRx technology can repress bladder cancer cell proliferation, trigger cell apoptosis, as well as reduce cell motility.

## Materials and Methods

### Patient Samples

To determine the expression of LINC00341 in tissue samples, we enrolled 36 patients diagnosed with urothelial carcinoma of the bladder who underwent partial or radical cystectomy into the study. Tumor histology was reviewed by two urological pathologists. The bladder cancer tissues along with the matched histologically non-tumor tissues acquired from the subjects were immediately snap-frozen in liquid nitrogen after surgical operation. All the subjects provided a written informed consent. Moreover, the Institutional Review Board of the Foshan Women and Children’s Hospital (Foshan, China) approved the study as per the declaration of Helsinki. subjects signed informed consents prior to sample collection.

### Cell Culture

To study the biological function of LINC00341 in cells, the T24 as well as 5,637 human bladder cancer cell lines were acquired from the Cell Bank of the Chinese Academy of Sciences (Shanghai, China). These cells were inoculated in minimal essential medium (DMEM) (Invitrogen, Carlsbad, CA, United States) enriched with 10% fetal bovine serum (Invitrogen, Carlsbad, CA, Unitd States) and allowed to grow. All media contained 1% penicillin/streptomycin. The culture environment was humidified air enriched with 5% CO_2_ at 37°C.

### Real-Time Quantitative PCR

To investigate the RNA expression of LINC00341, Trizol (Ambion, Austin, TX, United States) was employed to isolate total RNA as described by the manufacturer. RNA was reverted into complementary DNA (cDNA) using the Promega M-MLV kit. The sequences of the primers were: LINC00341 primers (Hu et al., 2014) forward: 5’- GCA​GGA​CTC​AGC​ATC​TCC​CA -3’, reverse: 5’- CTC​GGC​TGG​ACA​AGG​TGG​TT -3’; p21 primers forward: 5’- GGG​ATG​AGT​TGG​GAG​GAG​G -3’, reverse: 5’-CGG​CGT​TTG​GAG​TGG​TAG -3’; Bax primers forward: 5’- TGG​CAG​CTG​ACA​TGT​TTT​CTG​AC-3’, reverse: 5’- TCA​CCC​AAC​CAC​CCT​GGT​CTT-3’; E-cadherin primers forward: 5’- CGC​ATT​GCC​ACA​TAC​ACT​CT -3’, reverse: 5’- TTG​GCT​GAG​GAT​GGT​GTA​AG -3’; GAPDH primers forward: 5’-CGC​TCT​CTG​CTC​CTC​CTG​TTC-3’, reverse: 5’-ATC​CGT​TGA​CTC​CGA​CCT​TCA​C-3’. The PCR reaction volume of 20 μl consisted of 10 μl of 2 × All-in-OneTM qPCR Mix (GeneCopoiea Inc., Rockville, MD, United States), 0.4 μl ROX Reference Dye, 0.4 μl forward primer, 0.4 μl reverse primer, 1 μl First-Strand cDNA, as well as 7.8 μl DEPC treated water. The PCR was run and analyzed on an ABI PRISM 7000 Fluorescent Quantitative PCR System (Applied Biosystems, Foster City, CA, United States). Each PCR reaction was replicated thrice. GAPDH served as the internal standard. The PCR cycling conditions were: 10 min initial denaturation step at 95°C, 40 cycles constituting of 15 s denaturation at 95°C, 20 s annealing at 55°C, and 30 s extension at 70°C. In each triplicate, the median was employed to compute the relative LINC00341 level via the comparative ΔCt approach (value of 2-ΔCt (LINC00341- GAPDH)). After normalization with reference to GAPDH expression, expression fold changes were computed through the 2-ΔΔCt approach (VanGuilder et al., 2008).

### CRISPR-CasRx Plasmid Construction and sgRNA/siRNA Transfection

The sgRNA targeting LINC00341 has been designed, synthesized and inserted into the downstream of the U6 promoter, while the -CasRx gene was driven by the CMV promoter.

Genepharma Co., Ltd. (Suzhou, China) synthesized the p21 siRNA for the knockdown of gene expression. The p21 siRNA sequence (Sense) 5’- GAU​GGA​ACU​UCG​ACU​UUG​UUU-3’; (Antisense) 5’- ACA​AAG​UCG​AAG​UUC​CAU​CUU-3’. siRNAs targeting Bax along with E-cadherin were supplied by Genepharma (Suzhou, China). The negative control siRNA was also purchased from Genepharma Co., Ltd., Suzhou, China. Transfection of either the specific siRNAs or the siRNA negative control into the cell lines was done using the Lipofectamine 2000 Transfection system (Invitrogen, Carlsbad, CA, United States) as described by the manufacturer.

### Cell Counting Kit-8 (CCK-8) Assay

To explore the impact of LINC00341 on cell growth, the Cell Counting Kit-8, CCK-8 (Beyotime Institute of Biotechnology, shanghai, China) was employed to explore cell growth as per the protocol provided by the manufacturer. Cells were planted into 96-well plates and maintained overnight at 37°C. Afterwards, transfection of these cells with LINC00341 sgRNA/-CasRx or the negative control sgRNA/CasRx was conducted. At 0, 24, 48 as well as 72 h post-transfection, we introduced 10 μl of CCK-8 (5 mg/ml) into each well, allowed the cells to grow for an additional 1 h. After that, a microplate reader (Bio-Rad, Hercules, CA, Unites States) was employed to read the OD values of the samples at a wavelength of 450 nm. Finally, we converted the OD values into cell numbers via standard curves.

### Cell Apoptosis Assessment

To study the impact of LINC00341 on cell apoptosis, the caspase three ELISA assay kit (R&D, Minneapolis, MN, Unites States) was utilized to explore cell apoptosis as described by the manufacturer. The bladder cancer cells were grown and then transfection of either the LINC00341 sgRNA/-CasRx or the sgRNA/-CasRx negative control into cells was performed with the lipofectamine 2000 transfection system as per the manufacturer provided protocol. Thereafter, the cells were allowed to grow for 48 h, and then apoptosis triggered by LINC00341 knockdown was explored through exploring the caspase three activity as described by the manufacturer. Afterwards, the OD values were mesured with the microplate reader (Bio-Rad, Hercules, CA, Unites States). Data are indicated as the ratios of the OD values of LINC00341 sgRNA-transfected cells to the sgRNA negative control -transfected cells.

### Wound Healing Assessment

To study the impact of LINC00341 on cell migration, we cultured the bladder cancer cell lines and transfected them with either LINC00341 sgRNA or negative control sgRNA. Thereafter, a monolayer of the cells was scratched and then grown under normal parameters. The migration distances were finally determined at 0, 24 h after scratching for T24 and 5,637 cells, respectively.

### Tumorigenicity Assay in Nude Mice

Cells suspensions (5 × 10 7 cells/ml) were prepared using normal saline. We enrolled six 4-6-week-old male nude mice of specific-pathogen free (SPF) (weight: 18–20 g, an average of 18.81 ± 0.78 g) supplied by Shanghai SLAC Laboratory Animal Co., Ltd. (Shanghai, China). Following anesthesia with pentobarbital sodium, we routinely disinfected the nude mice followed by subcutaneous administration of 200 μl of tumor cell suspension. Afterwards, the mice underwent culture under SPF conditions.

### Western Blot Assay

To study the influence of LINC00341 on target gene expression, PBS was employed to rinse the T24 as well as 5,637 cells, followed by lysing with the RIPA buffer. Thereafter, quantitation of the proteins was performed via the BCA protein assay. An equivalent amount of the whole protein isolates were fractionated on an SDS-PAGE , and then blotting onto the PVDF membrane (Millipore, Billerica, MA, United States) performed. Subsequently, blocking of the samples was conducted using 5% dry milk. Thereafter, the samples were inoculated with the primary antibody against E-cadherin (1: 200; Santa Cruz Biotechnology, Santa Cruz, CA, United States), hBAX (1:1,000; Santa Cruz Biotechnology, Santa Cruz, CA, United states), as well as p21 (1:200; Santa Cruz Biotechnology, Santa Cruz, CA, United States), and GAPDH (1:10,000; Sigma-Aldrich) and incubated overnight. Afterwards, inoculation of the samples with HRP-labelled secondary antibodies (Amersham, Piscataway, NJ, United States) was performed, and Western blots were performed with the SuperSignal chemiluminescence reagents (Pierce Chemical Co.).

### Statistical Analysis

A paired sample t-test was employed to analyze the difference in LINC00341 RNA expression between bladder cancer tissue and matched non-tumor tissue. The independent samples t-test was employed to explore the differences in LINC00341 RNA expression between cancer subgroups. ANOVA was applied to examine the differences between the different groups in the CCK-8 analysis. Moreover, an independent sample t-test was utilized to analyze the apoptosis assay and wound healing assay. Pearson’s coefficient correlation is applied to measure the expression correlation. The SPSS (version 19.0 SPSS Inc.) as well as GraphPad Prism 8.0 (GraphPad Software, San Diego, CA, Unites States) softwares were employed to perform all the statistical analyses. *p* <0.05 signified statistical significance. All data are indicated as the mean ± SD.

## Results

### LINC00341 was Overexpressed in Bladder Cancer

**FIGURE 1:**
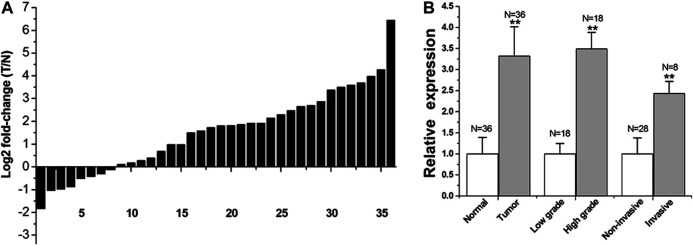
LINC00341 was overexpressed in bladder cancer.qRT-PCR was employed to explore LINC00341 relative expression. In each control group, the relative expression value of LINC00341 was set to a basic value of 1. N, the number of samples in each group. **(A)**. T stands for tumor and N stands for normal. The height of each column in the chart indicates the fold change (tumor/non-tumor) expressed by LINC00341 in 36 patients after log 2 conversion. **(B)**. Comparing the expression level of LINC00341 between tumor and healthy tissues (**p < 0.01). Differences in the expression of LINC00341 between high-grade cancer and low-grade cancer was compared (**p < 0.01). The expression level of LINC00341 in invasive cancer and non-invasive cancer was compared (**p < 0.01).

The relative LINC00341 expression in 36 bladder cancer patients was explored via qRT-PCR. Figure 1A shows the fold change in the expression of LINC00341 (bladder cancer tissue/corresponding histological non-tumor tissue) in every patient. Table 1 indicates the clinical features of this group of patients. In contrast with the matched non-tumor tissues, LINC00341 was overexpressed in bladder cancer (Figure 1B).

**TABLE 1:** Clinical characteristics of patients with bladder cancer.

No.	Sex	Age	Grade	Stage	Surgery	No.	Sex	Age	Grade	Stage	Surgery
1	M	56	Low	T2N0M0	Partial	19	M	60	Low	T1N0M0	Partial
2	M	68	Low	T1N0M0	Partial	20	M	63	High	T2N0M0	Radical
3	M	52	Low	T2N0M0	Partial	21	M	62	Low	T1N0M0	Partial
4	M	62	High	T3N0M0	Radical	22	M	53	High	T2N0M0	Radical
5	M	67	Low	T2N0M0	Partial	23	M	59	High	T2N0M0	Radical
6	M	56	Low	T1N0M0	Partial	24	M	49	High	T3N0M0	Radical
7	M	53	High	T2N0M0	Radical	25	M	41	High	T2N0M0	Radical
8	M	62	Low	T2N0M0	Partial	26	M	59	High	T2N0M0	Radical
9	M	42	Low	T1N0M0	Partial	27	M	57	High	T3N0M0	Radical
10	M	76	Low	T2N0M0	Partial	28	M	54	High	T3N0M0	Radical
11	M	55	High	T2N0M0	Radical	29	M	57	High	T3N0M0	Radical
12	M	55	High	T2N0M0	Radical	30	M	55	Low	T1N0M0	Partial
13	M	51	Low	T2N0M0	Partial	31	F	50	Low	T1N0M0	Partial
14	M	52	Low	T2N0M0	Partial	32	F	53	Low	T1N0M0	Partial
15	M	70	High	T2N0M0	Radical	33	F	50	Low	T1N0M0	Partial
16	M	54	Low	T1N0M0	Partial	34	F	51	High	T3N0M0	Radical
17	M	52	Low	T1N0M0	Partial	35	F	43	High	T3N0M0	Radical
18	M	58	High	T2N0M0	Radical	36	F	68	High	T3N0M0	Radical

No., patient number; M, Male; F, Female. Age, years old, Grade, the 2004 WHO classification. Stage, AJCC TNM classification. Radical, radical cystectomy. Partial, partial cystectomy.

We examined differences in expression based on grade and stage. The bar graph indicates the lncRNA-LINC00341 relative expression in all the groups. The LINC00341 expression level in high-grade tumors is higher in contrast with that in low-grade tumors (Figure 1B). The LINC00341 expression level in infiltrating tumors was higher relative to that in non-infiltrating tumors (Figure 1B).

### Specific sgRNA/-CasRx Down-Regulated LINC0034l Expression

The T24 as well as 5,637 bladder cancer cells were grown and inserted with LINC00341 sgRNA/-CasRx or negative control sgRNA/-CasRx. The RNA expression of LINC00341 was assayed 48 h after transfection via qRT-PCR. Consequently, the inhibition rate (LINC00341 sgRNA/negative control sgRNA) was 82.14 ± 2.67% and 78.31 ± 3.82% in T24 cells and 5,637 cells, respectively. The data are expressed as mean ± SD. Each sample was assayed in triplicates.

### Knockdown of LINC00341 by CRISPR-CasRx Repressed Cell Proliferation

**FIGURE 2:**
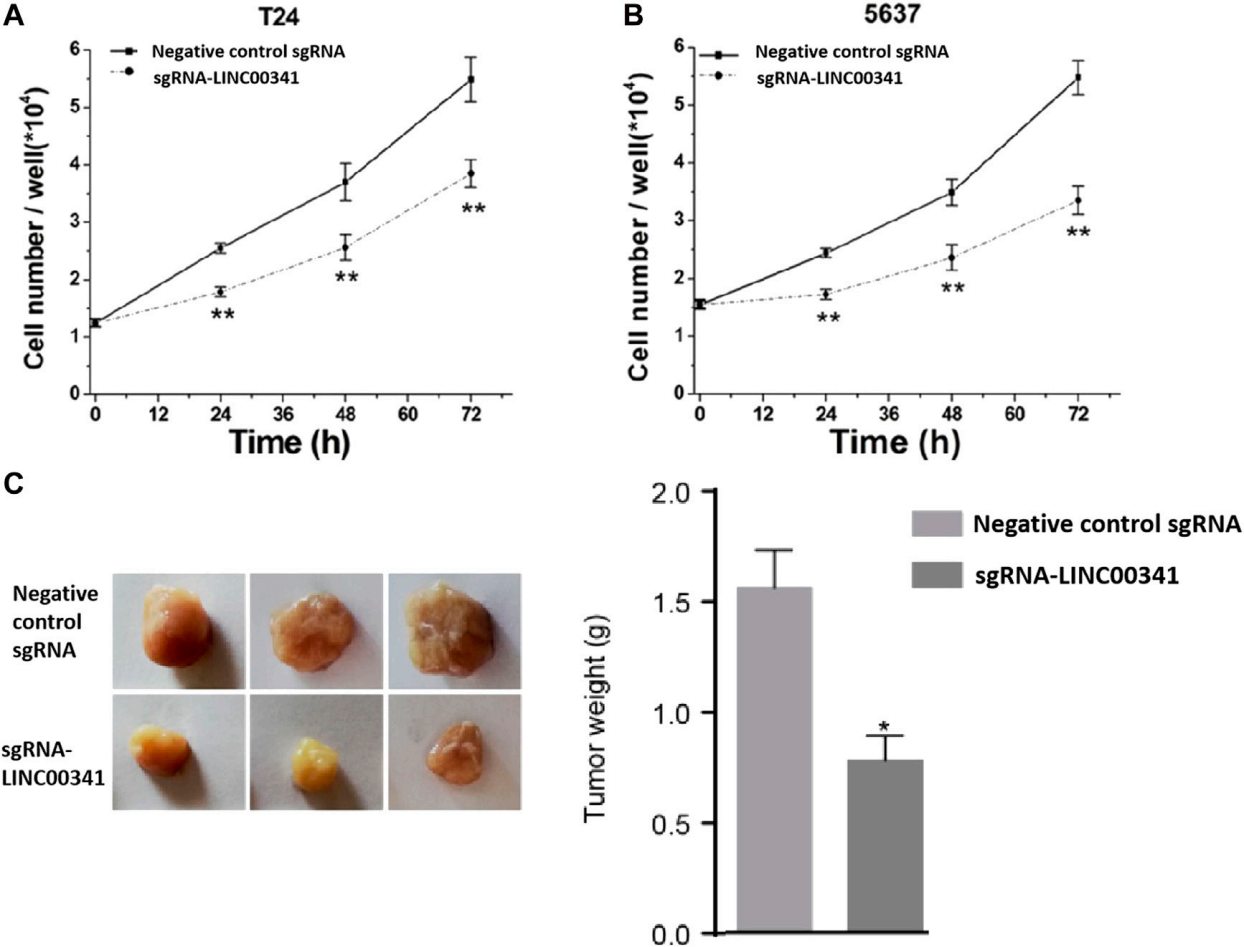
Knockdown of LINC00341 by CRISPR-CasRx suppressed cell proliferation. CCK-8 assay was employed to explore cell proliferation. After transfection with LINC00341 sgRNA or negative control sgRNA, the OD value was determined and converted into cell number. Analysis of variance is used to compare cell proliferation curves. **(A)**. There was suppression of cell proliferation in T24 bladder cancer cells (** *p* < 0.01). **(B)**. There was suppression of cell growth in 5,637 cells (**p < 0.01). The data is shown as the average ± SD. The experiments were conducted in triplicates. **(C)**. There was inhibition of tumor growth *in vivo* in 5,637 bladder cancer cells (**p < 0.01). The data is indicated as the average ± SD.

We transfected the T24 as well as 5,637 bladder cancer cells with LINC00341 sgRNA or negative control sgRNA, and then determined the rate of growth of bladder cancer cells proliferation via the CCK-8 assay. After knocking down LINC00341 (Figure 2B), the cell proliferation of T24 cells (Figure 2A) as well as 5,637 cells were inhibited. The inhibitory effect of LINC00341on the growth of tumors *in vivo* was also confirmed (Figure 2C), which indicated that LINC00341 repressed bladder cancer cell proliferation.

### Knockdown of LINC00341 by CRISPR-CasRx Induced Apoptosis

**FIGURE 3:**
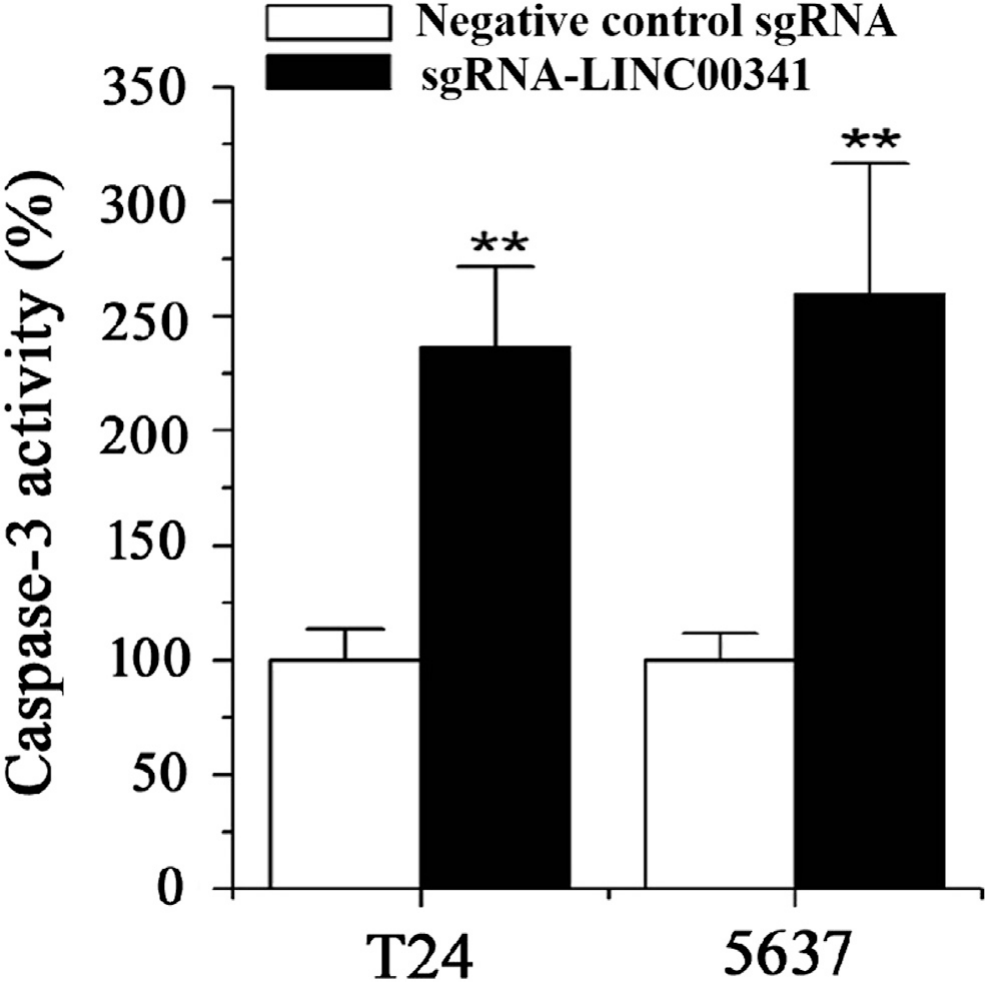
Knockdown of LINC00341 by CRISPR-CasRx triggered apoptosis. 48 h post transfection with LINC00341 sgRNA or the negative control sgRNA, the changes of apoptosis were measured by ELISA. Apoptosis induction was observed in T24 cells (**p < 0.01) and 5,637 cells (**p < 0.01) transfected with LINC00341 sgRNA via ELISA. The data is indicated as the average ± SD. The experiments were conducted in triplicates for three independent times for both cell lines.

Transfection of T24 as well as 5,637 bladder cancer cell lines with LINC00341 sgRNA or negative control sgRNA was performed. 48 h post transfection, we utilized ELISA to assay for the apoptosis of T24 as well as 5,637 bladder cancer cells (Figure 3). After knocking down LINC00341, induced apoptosis occurred in T24 as well as 5,637 bladder cancer cell lines.

### Motility Changes Triggered by Knockdown of LINC00341 Using CRISPR-CasRx

**FIGURE 4:**
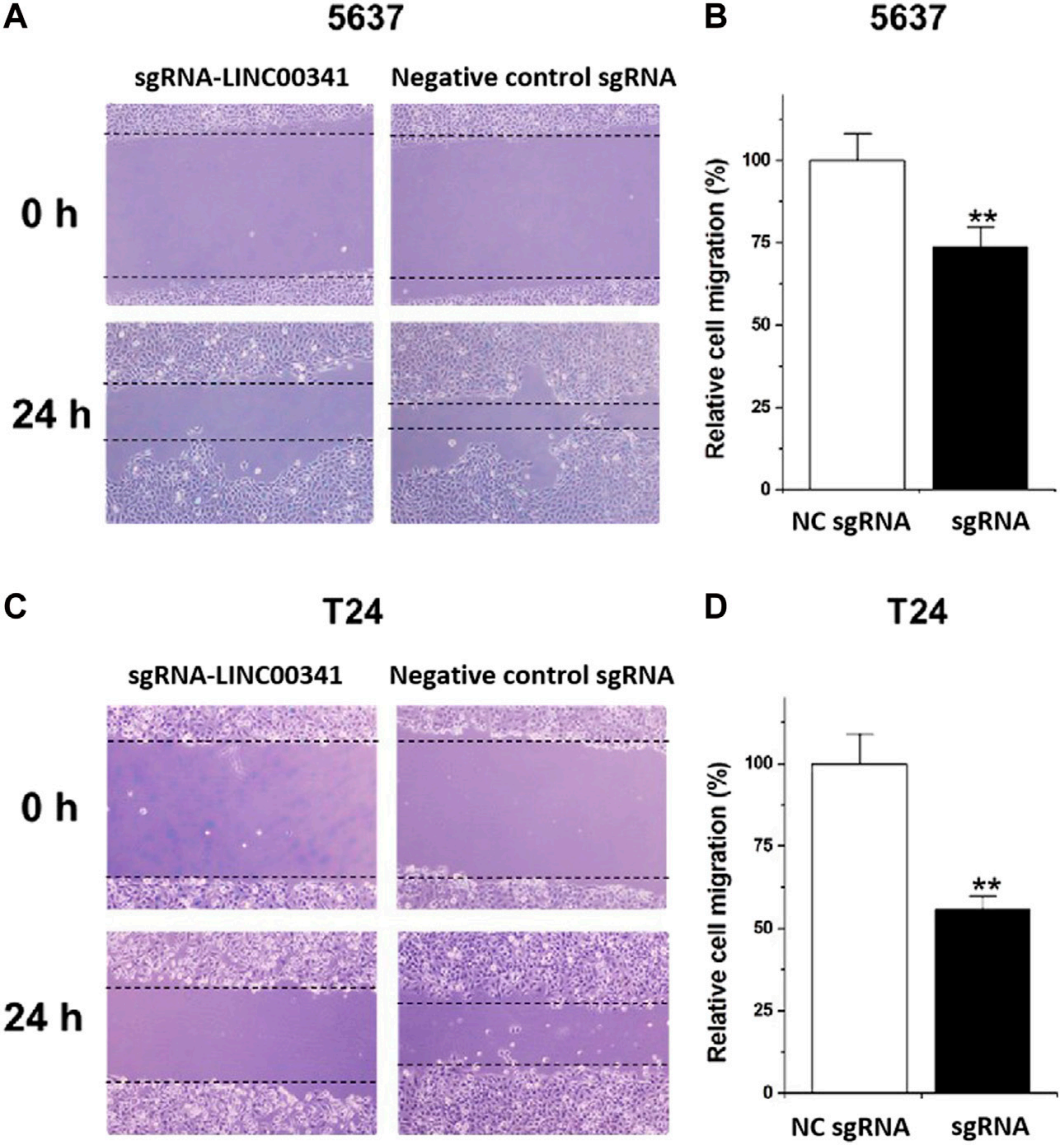
Knockdown of LINC00341 by CRISPR-CasRx decreased cell motility. Following the transfection with LINC00341 sgRNA or the negative control sgRNA, we performed the wound healing analysis to explore the cell motility alterations in T24 as well as 5,637 cells. **(A)**. Wound healing assay illustrative images in 5,637 cells. **(B)**. Diminished cell motility was seen in 5,637 cells (**p < 0.01). **(C)**. Wound healing assay illustrative images of T24 cells. **(D)**. Diminished cell motility occurred in T24 cells (**p < 0.01). Data are indicated as mean ± SD. The experiments were conducted in triplicates for three independent times for both cell lines.

We employed the wound healing to explore the changes in motility of the cells triggered by LINC00341 knockdown in bladder cancer cells. Diminished motility of the cells was reported in 5,637 cells as indicated in Figures 4A,B, as well as T24 cells as indicated in Figures 4C,D.

### Knockdown of LINC00341 by CRISPR-CasRx Increased p21, Bax as well as E-Cadherin Protein Expression

**FIGURE 5:**
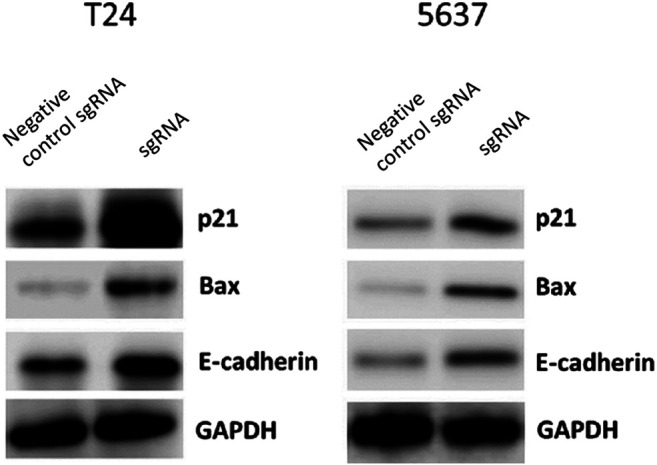
Knockdown of LINC00341 by CRISPR-CasRx increased p21, Bax and E-cadherin protein expression. LINC00341 sgRNA or negative control sgRNA transfected cells were subjected to western blot assay to examine expression changes of p21, E-cadherin, as well as Bax in bladder cancer cells. **(A)**. Western blot results showing protein expression T24 cells. **(B)**. Western blot assay images showing protein expression in 5,637 cells.

**FIGURE 6:**
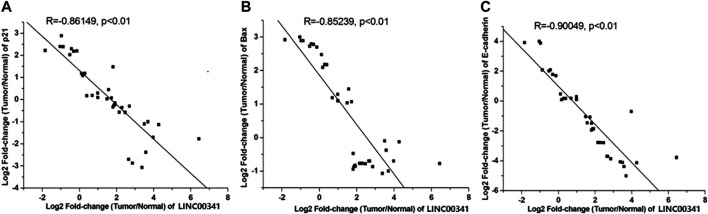
Negative correlation of LINC00341 with p21, Bax and E-cadherin expression. qPCR was employed to assay the expression changes of p21, E-cadherin, as well as Bax in bladder cancers compared to non-tumor tissues (N = 36). **(A)**. Inversely expressed LINC00341 and p21 in bladder cancer. **(B)**. Inversely expressed LINC00341 and Bax in bladder cancer. **(C)**. Inversely expressed LINC00341 and E-cadherin in bladder cancer.

**FIGURE 7:**
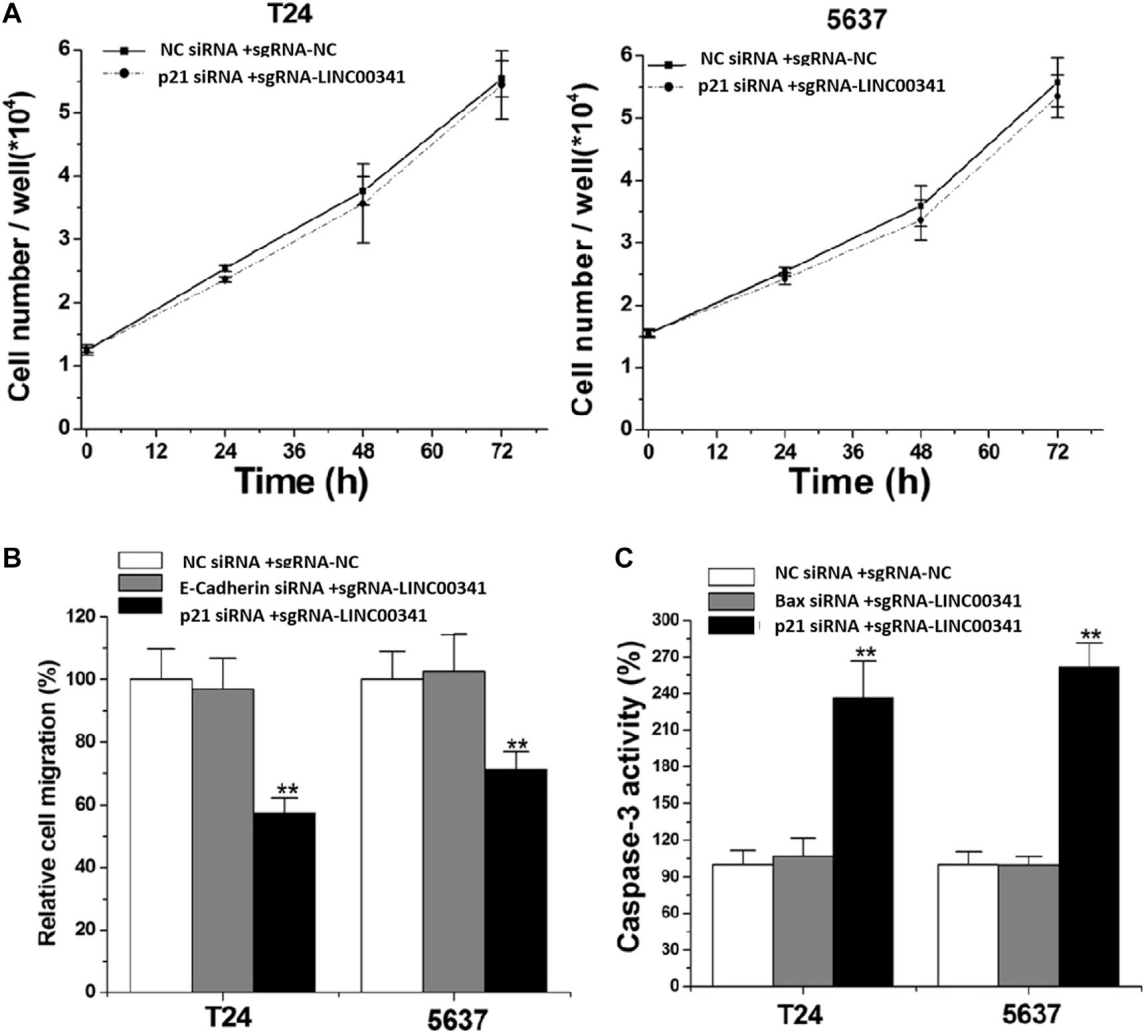
Knockdown of LINC00341 by CRISPR-CasRx induced cellular phenotypic changes via p21, Bax as well as E-cadherin regulation. After siRNA transfection, CCK-8, wound healing as well as ELISA analyses were respectively employed to detect cell proliferation, migration as well as apoptosis. **(A)**. p21 siRNA eliminated the proliferation inhibitory effect induced by LINC00341 sgRNA in T24 as well as 5,637 (*p* >.05). **(B)**. E-cadherin siRNA eliminates the migration inhibitory effect induced by LINC00341 sgRNA in T24 and 5,637, while p21 siRNA does not have this function. **p < 0.01. **(C)**. Bax siRNA eliminates the apoptosis-promoting effect induced by LINC00341 sgRNA in T24 and 5,637, while p21 siRNA does not have this function. **p < 0.01.

To assess the prospective biomarkers that trigger the aforementioned phenotypic alterations after knocking down LINC00341, Western blot was conducted to assess the protein contents of p21, Bax, as well as E-cadherin that have vital roles in the bladder cancer development. LINC00341 sgRNA significantly up-regulated the p21, Bax as well as E-cadherin expressions in T24 and 5,637 cells as indicated in Figures 5A,B at the protein level. In addition, clinical samples also showed that LINC00341 was negatively correlated with p21 (Figure 6A), Bax (Figure 6B) and E-cadherin expression (Figure 6C). To further verify the functional role of these markers in the LINC00341 knockdown experiment, we also used two siRNAs for double knockdown. p21 siRNA abolished the proliferation inhibitory effect induced by LINC00341 sgRNA in T24 and 5,637 (Figure 7A). E-cadherin siRNA abolished the migration inhibitory effects induced by LINC00341 sgRNA in T24 and 5,637 (Figure 7B). Bax siRNA eliminated the apoptosis-promoting effect induced by LINC00341 sgRNA in T24 and 5,637 (Figure 7C). p21 siRNA could not reverse the migration inhibitory effect (Figure 7B) and apoptosis promotion effect (Figure 7C) induced by LINC00341 sgRNA in these cells, possibly because p21 alone could not induce cell death or inhibit cell migration.

## Discussion

Recent studies have shown that human cancer is caused by a large number of lncRNA disorders. It is thought that lncRNA has great clinical significance as a set of cancer molecular markers as well as treatment targets(Sahu et al., 2015; Sun and Kraus, 2015). For example, it is reported that lncRNA PVT1 (Zhuang et al., 2015), SUMO1P3 (Zhan et al., 2016) and CCAT2 (Li et al., 2016) can facilitate cell proliferation as well as repress cell apoptosis in bladder cancer. As a recently discovered lncRNA, no work has been done to characterize the carcinogenic properties of LINC00341. It may provide the potential target of cancer treatment (Zhong et al., 2015).

LINC00341 may be a cancer driving factor in tumor development. Therefore, it is very interesting to explore its biological role in bladder cancer. For this purpose, herein, we established that LINC00341 is overexpressed in bladder cancer in contrast with the corresponding non-tumor tissues. The high expression of LINC00341 is related to high-grade as well as staged bladder cancer. The differential expression pattern of LINC00341 between bladder cancer and control along with the association of LINC00341 with clinicopathological characteristics indicate that the lnRNA LINC00341 has a new role in the occurrence and development of bladder cancer. To elucidate the possible influences of LINC00341 on bladder cancer, we assessed the changes in cell proliferation, apoptosis, as well as motility induced by LINC00341 silencing by CRISPR-CasRx in bladder cancer. Although traditional methods including siRNA/shRNA also offers a possibility for inhibiting LINC00341, the unexpected off-target effects significantly limit their applications. As a newly identified Cas system from RNA-targeting CRISPR enzymes, CRISPR-CasRx exhibits high efficiency and specificity for RNA cleavage (Jiang et al., 2020; Zhou et al., 2020). Inhibition of cell proliferation, escalated the rate of apoptosis, and reduced the motility rate in LINC00341 sgRNA-transfected T24 as well as 5,637 bladder cancer cell lines.

In order to study the potential ways to induce the above phenotype, we also performed a Western blot experiment and found that knocking down LINC00341 by CRISPR-CasRx can increase the expression of p21, Bax and E-cadherin. This finding is congruent with previous studies, which indicated that p21 is a downstream target of LINC00341. In other studies on bladder cancer, inactivation of p21 has also been shown to promote tumorigenesis and cell proliferation (Tang et al., 2015). Bax is a vital homologue of Bcl-2, an enhancer of apoptosis, and can be used as an independent parameter for predicting the clinical prognosis of individuals with bladder cancer (Golestani Eimani et al., 2014; Liu et al., 2016). E-cadherin is a cell-cell junction protein that is often absent during the migration of bladder cancer cells (Liu et al., 2014; Zhao et al., 2014). Using double knockdown experiments, we further show that the expression changes of these biomarkers can at least partially explain the phenotypic changes after LINC00341 knockdown. LINC00341 may be associated with an epigenetic repressor, thereby regulating the transcription of Bax or E-cadherin.

In summary, the above data indicate that LINC00341 serves as an oncogene in the carcinogenesis of bladder cancer. Targeting LINC00341 may be a promising method for human cancer gene therapy. More work is required to establish the prospective molecular mechanism of LINC00341 in the regulation of Bax as well as E-cadherin in bladder cancer. The treatment of LINC00341 should also be studied in depth.

## Data Availability

The original contributions presented in the study are included in the article/Supplementary material, further inquiries can be directed to the corresponding author.
